# BBMRI-ERIC’s contributions to research and knowledge exchange on COVID-19

**DOI:** 10.1038/s41431-020-0634-8

**Published:** 2020-05-22

**Authors:** Petr Holub, Lukasz Kozera, Francesco Florindi, Esther van Enckevort, Morris Swertz, Robert Reihs, Andrea Wutte, Dalibor Valík, Michaela Th. Mayrhofer, on behalf of the BBMRI-ERIC community

**Affiliations:** 1 BBMRI-ERIC, Neue Stiftingtalstraße 2/B/6, 8010 Graz, Austria; 2 Institute of Computer Science, Masaryk University, Botanická 68a, 60200 Brno, Czech Republic; 3 Department of Genetics CB50, University Medical Center Groningen & BBMRI-NL, Hanzeplein 1, 9713 GZ Groningen, Netherlands; 4 Medical University Graz & BBMRI.at, Auenbruggerpl. 2, 8036 Graz, Austria; 5 Masaryk Memorial Cancer Institute, Žlutý kopec 543/7, 602 00 Brno, Czech Republic

**Keywords:** Medical research, Social sciences

## Abstract

During the COVID-19 pandemic, the European biobanking infrastructure is in a unique position to preserve valuable biological material complemented with detailed data for future research purposes. Biobanks can be either integrated into healthcare, where preservation of the biological material is a fork in clinical routine diagnostics and medical treatment processes or they can also host prospective cohorts or material related to clinical trials. The paper discussed objectives of BBMRI-ERIC, the European research infrastructure established to facilitate access to quality-defined biological materials and data for research purposes, with respect to the COVID-19 crisis: (a) to collect information on available European as well as non-European COVID-19-relevant biobanking resources in BBMRI-ERIC Directory and to facilitate access to these via BBMRI-ERIC Negotiator platform; (b) to help harmonizing guidelines on how data and biological material is to be collected to maximize utility for future research, including large-scale data processing in artificial intelligence, by participating in activities such as COVID-19 Host Genetics Initiative; (c) to minimize risks for all involved parties dealing with (potentially) infectious material by developing recommendations and guidelines; (d) to provide a European-wide platform of exchange in relation to ethical, legal, and societal issues (ELSI) specific to the collection of biological material and data during the COVID-19 pandemic.

## Introduction

During the COVID-19 pandemic, the European biobanking infrastructure is in a unique position to preserve valuable biological material complemented with detailed data for future research purposes. Many biobanks are integrated into healthcare, where preservation of the biological material is a fork in clinical routine diagnostics and medical treatment processes; these are called clinical biobanks or clinical collections. Biobanks can also host prospective cohorts or material related to clinical trials. Amount and structure of collected data in clinical collections is determined by the specifics of the particular healthcare systems—typically based on the hospital information systems—for potentially large numbers of patients, especially when these are handled by the healthcare during pandemics. Prospective cohorts and clinical trials feature more structured data models and large amounts of data collected in controlled conditions, but at the cost of having a substantially lower number of patients.

Owing to the pandemic, we can observe an unprecedented stressing of the healthcare systems, which requires that, more than ever, sample and data collection happens as unobtrusively as possible for the primary care. Data collection processes, while they are dependent on local/national settings, should result in well-defined structured data suitable for multisite national and international data integration and large-scale machine analyses, following FAIR [1] and FAIR-Health [2] principles (extensions of FAIR related to quality and privacy). In addition, biobanking material with (possibly) infectious viral material should be prepared in relatively sparse BSL-3 certified laboratories; alternatively, material needs to be deactivated to enable processing in much more abundant BSL-2 laboratories.^1^

Amidst the COVID-19 crisis, the objectives of BBMRI-ERIC, the European research infrastructure established to facilitate access to quality-defined biological materials and data for research purposes, are clear: (a) to collect information on available European as well as non-European COVID-19-relevant biobanking resources and to facilitate access to these; (b) to help harmonizing guidelines on how data and biological material is to be collected to maximize utility for future research, including large-scale data processing in artificial intelligence; (c) to minimize risks for all involved parties dealing with (potentially) infectious material; (d) to provide a European-wide platform of exchange in relation to ethical, legal, and societal issues (ELSI) specific to the collection of biological material and data during the COVID-19 pandemic. Achieving these objectives will substantially support further research into COVID-19, and hence it is key to connect early and effectively in a multidisciplinary manner with other key facilitators of medical research, such as the research infrastructures ECRIN (multinational clinical trials) and EATRIS (translational medicine)^2^ or the broader community of research infrastructures, which already mobilized considerable resources across all scientific clusters.^3^

## COVID-19 resources in the BBMRI-ERIC network

In order to ensure findability of biobanking resources, BBMRI-ERIC operates two main services: (1) the BBMRI-ERIC Directory,^4^ which works on aggregate metadata descriptors of biobanks and their collections, and (2) the BBMRI-ERIC Locator,^5^ which works as a federated search tool allowing to query individual-level data at the sites of origin while returning aggregated results.

To date, the Directory has been at the center of BBMRI-ERIC’s initial COVID-19-related activities. The aggregated nature of the underlying data allows for a low entry bar to register resources and thus any COVID-19-related resources could be included easily regardless of the country of origin. The Directory data model is based on MIABIS Core 2.0 [3] with the concept of biobanks (institutions) and collections (of data and samples) and it has been extended with COVID-relevant attributes as shown in Table 1. Existing collections of COVID-positive samples will be marked with respective ICD-10 codes and COVID-specific collection attributes. Prospective collections can be advertised as empty collections with COVID-specific collection attributes defining what data can be collected. The metadata descriptors were initially collected by BBMRI-ERIC’s National Nodes (represented throughout 21 member states),^6^ and now the Directory is ready to accept updates via National Nodes or via the BBMRI-ERIC Helpdesk.^7^ The Directory data model is being continuously optimized based on the feedback from and needs of the research and biobanking communities. The model is also being deployed in national nodes to collect the relevant information, using the underlying MOLGENIS platform [4] to quickly configure locally needed extensions. In order to maximize findability, the Directory also serves as a data source for aggregation services such as FAIRSharing.org and Google dataset search.

**Table 1:** Overview of COVID-specific attributes added to BBMRI-ERIC Directory.

Attributes added on the biobank level	Attributes added on the collection level
Biobank providing services relevant to COVID-19: • Member COVID-19 network Availability of the services provided by the biobank: • Antibody development • Proteomics studies including protein engineering and protein interactions • Screening tools for searching virus proteases inhibitors • Animal testing facility • Ability to set up prospective collections • Ability to set up clinical trials • Virus sequencing facility • BSL-2 laboratory available • BSL-3 laboratory available • Laboratories doing PCR-based diagnosis	Relevant diagnoses: • U07.1—laboratory-confirmed COVID-19 diagnosis • U07.2—suspect or probable COVID-19 diagnosis Availability of relevant data and products: • Data on clinical symptoms • Data on disease duration and disease outcome • Antibodies titer (IgM and IgG) • CT imaging of lungs, alternatively Xray • Blood count and other lab results especially at the moment of hospital admission, • Treatment protocol (types of drugs used)

Effective access to the biobanking resources, as advertised in the Directory, is possible via the BBMRI-ERIC Negotiator^8^. After a researcher identifies relevant biobanks in the Directory, she can use the “Request access” functionality and follow the access procedure; this involves authenticating using BBMRI-ERIC Authentication and Authorization Infrastructure, describing her project and specific request details and information on ethics review if available. Once this initial information is provided, the identified biobanks are notified and the access negotiation starts. Using the Negotiator, the researcher can effectively negotiate with several biobanks at once and insufficiently specified requests can be quickly clarified.

In the near future, the federated search in the BBMRI-ERIC Locator will be extended with the COVID-specific attributes; the extension depends on an initial consensus of the international communities on common data models describing patient-level information. The BBMRI-ERIC community therefore participates in the global ‘COVID-19 Host Genetics Initiative’^9^ and actively monitors other initiatives such as OHDSI COVID-19 work.^10^ Outcomes of these initiatives will need to be practically assessed in order to ultimately verify what information can be effectively made available for querying in practice of clinical biobanking in different countries.

## Development of quality and safety guidance

Collection of quality biological material from patients with suspected and confirmed COVID-19 might be a challenge due to infection risk related to the procedures performed on different types of biospecimen. In most cases, samples collected from upper respiratory tract (swabs, sputum, bronchoalveolar lavage) seem to be associated with the highest risk due to the content of active virus when compared with the whole blood and blood derivatives [5]. In addition, assessment of the virus load in such samples collected from asymptomatic individuals and those who show typical COVID-19 symptoms, showed no statistically significant difference with the highest viral load at the presentation [6]. Stool samples, collected by many biobanks, are also amongst highly infectious material in contrast to serum and plasma [5, 7]. Safety measures for fractionation and storage of urine samples should be also performed due to inconsistent data on viral load, indicating that urine might be potentially infectious [8] but this stays in contrast with other reports [5]. While the most current publications are a valuable source of the latest knowledge, they might also provide conflicting advice and limited data. Laboratories performing work on biological specimens should thus strictly follow well-established guidelines such as those published by WHO,^11^ ECDC,^12^ as well as local epidemiological surveillance. According to these recommendations the individual risk assessment is becoming a key step before starting the work with SARS-CoV-2 suspected samples in order to decide whether sample processing requires BSL-3 or BSL-2 standard. High virulence of SARS-CoV-2 and the lack of suitable equipment in many facilities, indicates the possibility of taking steps that minimize contact with potentially infectious material. It is essential to establish the flow of biological material between clinical laboratories and biobanks that will perform the minimum steps associated with the processing of biological material and will be responsible for its safe storage until release—such as guidelines established by Czech National Node of BBMRI-ERIC [9]. In response to the needs of the biobanking community, BBMRI-ERIC organized a series of online meetings with clinical and laboratory experts and provided the access to guidelines enabling the preparation of biobanks for working with samples obtained from COVID-19 patients^13^. International standards on risk management and safety, such as ISO 20387:2018, 31000:2018, 31004:2013, and 15190:2020, should be also followed when applicable.

## Appropriate ethical and legal requirements

As regards to ethical requirements and biobanking, the same guidelines persist also in the context of COVID-19, allowing for practical adjustments in emergency situations.^14^,^15^,^16^ Oversight in the midst of the pandemic, typically the responsibility of the local ethics committee, has been frequently adjusted to meet the urgency of COVID-19 by ensuring fast response such as 48 h.

In relation to data processing during an emergency situation, the GDPR explicitly enables competent public health authorities to lawfully process personal data for reasons of substantial public interest in the area of public health conditionally without informed consent.^17^ In a guidance document on the management of clinical trials during the COVID-19 pandemic,^18^ for instance, it is further specified to obtain the consent from the patient (or her/his legal representative) at a later stage. This recommendation applies to “acute life-threatening situations”, where it is not possible to obtain consent in advance and only when allowed within national legislation. In the context of biobanks, broad consent can be used if it has already been integrated prior to the emergency situation in the clinical admission processes.

## Conclusion

By integrating the COVID-19 resources in the Directory, BBMRI-ERIC aims to contribute its unique European research infrastructure, with its National Nodes and collaborators not only the preservation of valuable biological material complemented with detailed data, but also to ensure timely knowledge exchange on quality management and guidance on safety and ethical and legal requirements and their practical application. Where appropriate in collaboration with other organizations or societies, BBMRI-ERIC will continue to organize and disseminate responses to the needs of the community, including webinars or FAQs.

## Footnotes

1 https://www.who.int/docs/default-source/coronaviruse/laboratory-biosafety-novel-coronavirus-version-1-1.pdf?sfvrsn=912a9847_2

2 https://eatris.eu/insights/bbmri-ecrin-and-eatris-join-forces-to-offer-covid-19-fast-response-service/

3 https://www.esfri.eu/covid-19

4 https://directory.bbmri-eric.eu/

5 https://samplelocator.bbmri.de/

6 https://www.bbmri-eric.eu/national-nodes/

7 directory-data@helpdesk.bbmri-eric.eu

8 https://negotiator.bbmri-eric.eu/

9 https://www.covid19hg.org and https://www.icda.bio/

10 https://www.ohdsi.org/covid-19-updates/

11 https://www.who.int/emergencies/diseases/novel-coronavirus-2019/technical-guidance

12 https://www.ecdc.europa.eu/en/covid-19-pandemic

13 https://www.bbmri-eric.eu/services/bbmriqm-covid

14 https://www.bbmri.it/en/nodo-nazionale/elsi-covid-19/assetto-etico-normativo/ (for an excellent overview of ELSI guidelines, tailored for the situation in Italy)

15 https://unesdoc.unesco.org/ark:/48223/pf0000146180 (esp Article 8. Respect for human vulnerability and personal integrity).

16 https://www.wma.net/policies-post/wma-declaration-of-helsinki-ethical-principles-for-medical-research-involving-human-subjects/ (esp principle 30, which is concerned with research participants that are mentally or physically unable to consent).

17 https://edpb.europa.eu/sites/edpb/files/files/file1/edpb_statement_2020_processingpersonaldataandcovid-19_en.pdf

18 https://ec.europa.eu/health/sites/health/files/files/eudralex/vol-10/guidanceclinicaltrials_covid19_en.pdf
